# Inter- and intra- individual variability in audiovisual multisensory processes in healthy ageing

**DOI:** 10.1016/j.nbas.2026.100168

**Published:** 2026-09-14

**Authors:** Naomi K. Middelmann, Lauriane Ecabert, Eduardo Villar Ortega, Alessandra Griffa, Andrea Brioschi-Guevara, Gilles Allali, James E. Bartlett, Micah M. Murray

**Affiliations:** a The Radiology Department, Lausanne University Hospital and University of Lausanne, Switzerland; b The Sense Innovation and Research Center, Lausanne, and Sion, Switzerland; c Child and Adolescent Psychiatry Service, Department of Psychiatry, Lausanne University Hospital and University of Lausanne, Switzerland; d The Leenaards Memory Center, Department of Clinical Neurosciences, Lausanne University Hospital and University of Lausanne, Switzerland; e School of Psychology and Neuroscience, University of Glasgow, Glasgow, United Kingdom

**Keywords:** Ageing, Multisensory, Crossmodal, Auditory, Visual, Reaction time, Variability

## Abstract

With an increasingly large segment of the population facing age-related cognitive impairment, understanding subtle changes before cognitive decline is crucial. Typically, sensory processing in ageing has been studied in isolation, and studies of multisensory processes have yielded mixed results. Furthermore, studies of reaction times (RTs) have often focused on aggregate measures, while a growing body of work shows the relevance of characterising intra-individual variability as a precursor to cognitive decline and fall risk. Lastly, changes in auditory processing of pitch have also been linked to cognitive decline. There is thus an imperative to identify cost-effective and accessible screening tools for assessing sensory and cognitive (dys)functions, particularly with ageing populations that may be reluctant to visit clinics or undergo brain imaging or extensive cognitive testing. Here, **55 cognitively healthy, older participants (aged 60–84 years) completed a simple detection task with auditory, visual, and simultaneous audiovisual multisensory stimuli. RTs to multisensory stimuli were significantly faster and less variable than those to either visual or auditory stimuli, and those to auditory stimuli were slowest and most variable. This speeding exceeded predictions based on probability summation, suggestive of integration prior to initiating motor responses. Further analyses revealed RT differences between auditory stimuli according to their pitch. On the one hand, the results underscore how multisensory contexts can improve stimulus processing during healthy ageing. On the other hand, the results highlight sensitivity to age-related changes in auditory sensory processing. Collectively, the results support detection tasks as a promising assessment of age-related changes in sensory processing.**

## 1 Introduction

Worldwide, populations are rapidly ageing with the number of people over 65 years old set to increase two-fold by 2050 [1]. This demographic shift has led to a higher proportion of society suffering from cognitive decline and dementia [2]. This is not only a significant health concern, but also a significant mental health concern as cognitive decline has been shown to lead to social isolation, stress, anxiety, and depression, which increases the risk of further cognitive impairment [3]. The effects of cognitive impairment extend far beyond memory issues, also affecting sensory perception (e.g., seeing and hearing), attention, and the ability to carry out tasks that require quick decision-making, such as safely crossing a street or driving [4]. Although much focus has been placed on understanding and assessing patients with cognitive impairment, exploring how ageing affects sensory processing in older adults without cognitive impairment is also crucial in order to understand the underlying processes of ageing as well as to potentially identify preventative measures that could delay the onset of cognitive decline [5], [6].

Even in the absence of diagnosed cognitive impairment, normal ageing is associated with a host of neural changes that alter how the brain encodes and integrates information across visual and auditory sensory modalities [4], [7], [8], [9], [10]. Emerging evidence suggests that older adults show changes in sensory perception and attentional modulation that can influence how sensory information is processed and integrated, leading to altered perceptual outcomes and increased reliance on compensatory strategies [11], [12]. Furthermore, research has suggested that even healthy ageing can be accompanied by modifications in the efficiency, timing, and coordination of neural processes that underlie perception, attention, and control [4], [6].

Vision changes are well-documented in adults over 60 [13], [14]. Older adults experience both reduced visual accuracy and precision in visual spatial information processing and a shrinking field of view [15]. While there are changes to the retina and cornea, there is more importantly a decline in the ability to integrate and process visual information [15]. These factors can impact navigation abilities or locating objects (such as one's phone or keys) in a cluttered space [15]. While visual processing changes during healthy ageing, auditory processing has likewise been shown to be heavily affected [16].

Auditory processing, even among those without presbycusis (age-related hearing loss), appears to decline more than the perception and integration of visual stimuli [16]. Research has shown changes to the central auditory processing system, as well as changes in memory and attention that can, for example, affect the ability to discern speech [13], [16]. Moreover, research suggests that modifications to the central auditory system affect the brain's ability to process interaural temporal and level differences, which are crucial sensory cues for locating sounds in space [13], [16], [17]. One hypothesis is that ageing brains have greater trouble filtering out irrelevant information particularly in noisy environments [17]. This decline in auditory processing capabilities can affect cognition and appear to be associated with an increased risk of dementia [18], [19], [20]. Whereas traditional auditory tests depend on an audiogram exam where patients are asked to signal when different sounds are detected, such tests do not measure response time differences to different sounds. However, it is well documented that detecting and treating auditory processing changes as early as possible is crucial to reducing risks of cognitive decline [21]. The need for early detection has led researchers to look for alternative ways to measure these subtle processing changes.

Declines or instability in pitch processing have been associated with broader auditory temporal deficits, subtle neural changes in temporal and prefrontal systems, and diminished attentional modulation—all of which can reflect early neural inefficiencies linked to ageing [22]. Moreover, pitch perception and auditory processing difficulties have been proposed as “neuromarkers” for cognitive decline, including prodromal stages of Alzheimer's disease [14], [16], [17], [19]. There is some evidence of faster reaction times to higher-pitch than lower-pitch tones in the context of a selective attention task completed by healthy young adults [23], though other studies involving a simple detection task reported no statistically reliable differences [24]. To the best of our knowledge, no study has investigated these possible detection speed differences in healthy ageing using a simple detection task. This is a gap in the literature, as evidence suggests that hearing loss in older adults, typically affects high-frequency more than low-frequency sounds [25]. Therefore, finding ways to assess and evaluate changes in auditory processing, including pitch, using simple measures is important. It is similarly important that such assessments capture not only accuracy (which is often emphasized in audiological examinations) but also speed and variability.

While unisensory processing, such as auditory or visual processing, and the effects of its impairment have been described, multisensory processing—the integration of information across visual, auditory, and somatosensory channels—represents a crucial, yet vulnerable, domain in ageing due to changes at a neurobiological level [9]. In multisensory situations (when the brain is treating information using two or more sensory inputs), it appears that behaviour is oftentimes quicker, more accurate, and less variable [26]. Efficient multisensory integration supports fundamental cognitive and behavioural functions, from speech comprehension and spatial orientation to motor coordination and social communication [26].

Whereas in studies of younger adults and schoolchildren, multisensory stimuli have clearly been shown to be beneficial and to lead to quicker detection times than auditory or visual stimuli alone [27], findings in older adults have been more mixed. Some research on older populations points to benefits from multisensory stimuli [8]. Other research has shown little or no benefit [12]. Still others report larger multisensory benefits in the elderly versus young [28], [29], In a study comparing auditory, visual and multisensory stimuli detection times in young adults and older participants with or without mild cognitive impairment, multisensory benefits were observed in all three groups [8]. Differences in results could stem from task complexity or paradigm design. For example, Peiffer et al. [29] and Murray et al. [8] used a simple detection task in their studies, Laurienti et al. [28] used a Go/NoGo 2-alternative forced choice task, and Atkin et al. [30] used multisensory stimuli in a lexical recall task that arguably required other cognitive processes such as working memory.

Understanding the extent and limits of multisensory integration and unisensory processing in ageing populations could allow for early intervention to help maintain cognitive health and design safer environments for older adults [31]. Murray et al. [8] suggested that detection time differences could be used to detect cognitive decline in older adults. In their study of 51 young participants, 49 healthy older participants and 21 participants with mild cognitive impairment, they were able to classify between older adults with and without impairment by applying a receiver operating characteristic (ROC) analysis, suggesting that simple detection tasks could be used as an assessment tool [8]. Although simple detection tasks can be effective for exploring the question of sensory perception comparing one population to another, to date they have not been used to look at the question of intra-individual variability (IIV or variability within a same individual) despite growing research on the importance of considering IIV in ageing [32], [33].

Studies using simple detection tasks have traditionally used aggregate metrics and compared median, mean and interquartile ranges across trials for their comparisons either across conditions and/or across populations (e.g. [8], [29]). However, a growing research field has shown the importance of considering IIV to explore how an individual's attention and performance vary over time and across modalities [32], [34], [35], [36]. Trial-to-trial variations in behavioural or neural responses within and across individuals is considered a sensitive index of attentional control and provides a means of monitoring an individual's sensory processing over time [34], [37], [38], [39]. From a neural standpoint, increased variability may arise from reduced signal-to-noise ratios in cortical processing or altered synchronization [38], [39], [40]. These shifts may underlie both increased cognitive load and subtle fluctuations in performance that manifest as IIV [41]. It could also signal inconsistencies in executive monitoring and the dynamic allocation of attentional resources [42]. Although IIV is a normal trait in all individuals, elevated IIV in older adults is thought to reflect transient lapses in sustained attention, diminished top-down regulation, or inefficient neural communication [41], [43], [44], [45], [46]. Furthermore, research has suggested that IIV may precede measurable cognitive decline, positioning it as a potential early marker of attentional difficulties in otherwise healthy ageing [41], which not only leads to a higher risk of falls, but also a greater risk for psychiatric illness [37]. These changes in processing and attentional control have been studied in how older adults perform on complex cognitive tasks, which could be affected by task comprehension issues and memory, or that depend the integrity of executive functions [41], [47]. Whereas IIV on unisensory and multisensory detection tasks has been researched in children and young adults [48], this has not yet been investigated in older adults. Examining IIV in older adults during a simple detection task using auditory, visual, and audiovisual stimuli could clarify how age alters sensory processing and attention and how age affects everyday perceptual functioning and risk-related outcomes.

Therefore, the present study, using a simple reaction time task in cognitively healthy older adults, expands beyond the existing literature by exploring reaction times to auditory, visual and audiovisual multisensory stimuli across multiple trials. First, it uses a more complex set of stimuli (two auditory and two visual stimuli) than that of Murray et al. [8] or Peiffer et al. [29] in order to observe how low-level stimulus features, such as auditory pitch, may contribute to the pattern of results in healthy older participants seen by Murray et al. [8]. This will include assessing whether reaction times to multisensory stimuli differ from those to visual or auditory stimuli alone. Second, it will assess IIV across trials as a function of sensory condition to explore attentional differences over time within individuals. Finally, it will compare reaction times for detection of higher and lower pitch sounds**.** Collectively, by examining response times and their variability to auditory, visual and multisensory stimuli across trials, across participants and within participants, this study will extend knowledge on sensory processing in older adults [7].

Specifically, we address three research questions and hypotheses. First, we investigate whether the multisensory benefit on simple reaction times seen in an elderly population [8], [29] replicates under similar methodological conditions involving a more varied stimulus set and more appropriate statistical modelling. We hypothesise that participants will demonstrate faster mean and median RTs to audiovisual multisensory stimuli than to either auditory or visual stimuli alone. Second, we characterise how IIV on this task is impacted by sensory modality and their combination. We hypothesise that multisensory conditions will reduce IIV compared with unisensory conditions, which would be consistent with patterns observed in young adults [49] and children [48]. Finally, in an exploratory analysis we will test whether the pitch range of the auditory stimulus impacts reaction times in healthy elderly adults. To the extent that older participants included in this study have no presbycusis, we hypothesise that RTs to higher-pitched sounds will be significantly faster than those to lower-pitched sounds.

## 2 Methods

The data analysed in this study are part of a larger research project and dataset, “Impact of Art Making for Healthy Ageing, Cognition, and Brain Function” (IAM4HA). The protocol received approval from the state ethics board (“Commission cantonale d'éthique de la recherche sur l'être humain CER-VD; protocol number: 2024–00548). The IAM4HA project examines the effects of 12 weeks of art or music theory training versus a waitlist control group in cognitively healthy participants aged 60 years and older. The full IAM4HA protocol includes MRI and fMRI data, neuropsychological assessments, a speech-in-noise test [50], eye-tracking, a drawing from perception and a drawing from memory task, as well as the simple detection task analysed here. Data are being collected at 2 timepoints: at recruitment (T1) and after the 12 weeks of classes (T2). Only data from T1 from the initial 55 participants, who completed T1 are considered here.

### 2.1 Participants

We recruited 55 cognitively healthy participants, aged 60–84 years. The mean age of participants was 68 years (SD = 5.14; Mdn =67), with 38 women and 17 men. The participants were recruited through flyers, social media, word of mouth and through the Swiss Brain Health Registry (BHR) database (https://www.bhr-suisse.org). All participants provided written informed consent to procedures approved by the cantonal ethics committee (protocol #2024–00584).

The inclusion criteria were 60 years or older with a Montreal Cognitive Assessment (MoCA) of ≥26 [51] and a Clinical Dementia Rating of 0, French language proficiency, and agreement to be notified in case of incidental findings. The exclusion criteria were a contraindication to MRI, training and/or experience in visual arts or music, history of head trauma or other neurological events, history of alcohol or substance abuse, uncorrected hearing or visual impairment, or an incapacity of discernment. All included participants had corrected-to-normal vision. All reported normal hearing, and none wore a hearing aid. Of the 55 participants, 85% were native French speakers. The others were proficient in French. Four of the participants had a high school diploma, 31 had a bachelor's degree, and 20 had a post-graduate diploma.

### 2.2 Materials

For the detection task, participants were presented with either visual, auditory or multisensory (simultaneous audiovisual) stimuli (hereafter V, A, and AV, respectively) and were asked to press the spacebar on a computer keyboard as quickly as they could detect a stimulus (see procedures below). Auditory stimuli were tones (44,100 Hz digitization; 16 bit stereo), and visual stimuli were either a “star” or “cloud” shape. The visual stimuli were uniformly white and centrally presented on a black background, subtending ∼5° of visual angle in both directions. The auditory stimuli were delivered bilaterally via the laptop's internal speakers and were perceived as centrally located. The tones differed in their spectral composition (see Supplementary Fig. S1). The “yawn” stimulus had peak energy in lower frequencies (i.e. <500 Hz), whereas the “whistle” had peak energy in higher frequences (i.e. ∼2000 Hz). The intensity of the auditory stimuli was measured using a CESVA SC-L sound pressure meter (https://www.cesva.com). The intensity at the ear of the “yawn” stimulus was 76 dB, and the intensity of the “whistle” was 80 dB. The AV conditions were any of the 4 combinations of the individual auditory and visual stimuli and were each equiprobable as detailed below. The order of the stimuli was varied to preserve a high level of attention and a certain unpredictability.

### 2.3 Procedure

After providing informed consent and collecting basic demographic data, including age and sex, participants were contacted by phone to set up three appointments according to their availability. A first meeting was set up with a licensed neuropsychologist at the Lausanne University Hospital (CHUV) who conducted neuropsychological assessments. A second appointment was set up on a different day for the psychophysics tests (described above), and a third appointment was set up for the MRI component.

On the appointment day for the psychophysics test, individual participants were welcomed to the laboratory at the CHUV by a staff member. There was only one participant at a time in the lab. The participant was then directed to sit in a comfortable desk chair in a sound-attenuated laboratory room. Researchers ensured that participants were positioned 60 cm from the laptop screen with a keyboard on the desk in front of them. Participants were explained the detection task and had the opportunity to ask questions if necessary. Participants were instructed to press the spacebar on the keyboard upon detection of a stimulus (A, V, or AV) as quickly as possible regardless of the stimuli presented. The researcher then stood outside of the lab room, while participants completed the simple reaction time task.

Participants completed a total of 120 trials, with each stimulus condition being equiprobable (i.e., 40 trials per A, V, and AV stimulus condition). Among the auditory trials, there were 20 presentations of the “yawn” and 20 presentations of the “whistle”. Among the visual trials, there were 20 presentations of the “star” and 20 presentations of the “cloud”. Among the audiovisual trials, there were 10 presentations of each combination of “yawn” +”star”, “yawn” + “cloud”,”whistle” + “star”, and “whistle” + “cloud”. The block of trials began with a visually presented countdown (i.e., 3, 2, 1) to inform the participant that the experiment would be beginning. Afterwards, there was a central fixation (white dot on a black background) continually present on the laptop's screen. Stimulus duration was 0.5 s, and the inter-stimulus interval (i.e. offset to onset timing) ranged from 1.5 to 1.9 s in steps of 0.1 s that were randomized across trials. This task took approximately 4.5 min to complete. Breaks were provided to participants between different tasks. Stimulus delivery and response collection were controlled by Psychopy version 2024.2.4 [52].

### 2.4 Statistical analyses

Although simple detection tasks are widely used and validated in the literature as providing robust measures of RTs, there remains ongoing debate regarding the most appropriate statistical methods for their analysis (e.g., [53]). RT data from a given participant are often positively skewed and non-normal [53], [54] (see also Supplementary Fig. S2). Some studies have relied on traditional aggregate measures, such as the mean, median, and IQR from each participant that are then submitted to a repeated measures analysis of variance (rm-ANOVA) [8] or on linear mixed effect models [55]. While these measures and analyses provide a summary of the data, Murray et al. [8] assumed normality of the distribution, and aggregated the data into one datum per participant.

Furthermore, descriptives do not fully account for IIV across trials or sensory modalities. Research has shown, for example, that RTs can exhibit priming effects over time, and individual differences in attention and response speed can influence results [54]. Simply reporting (and analysing) the mean or median does not capture these dynamic changes across trials. Furthermore, Brown [53] argues against using traditional ANOVA models, which, despite being robust, are not suitable for repeated measures data like RTs, where participants complete multiple trials. In designs that have repeated measures, RTs are correlated within participants, violating the assumption of independence required by ANOVA, which the analyses by Murray et al. [8] likewise overlooked. Additionally, ANOVA requires list-wise deletion, which can reduce sample size and lead to biased estimates [53]. Consequently, here we used generalised linear mixed-effects models (GLMMs), which accommodate non-normal data, non-aggregated data, and multiple data points per participant, as is the case in our detection task [54]. Furthermore, they are particularly well-suited to repeated measures over time, as they account for both fixed and random effects [54]. Additionally, GLMMs can model the variability within participants and across trials [56]. This is particularly important in reaction time research where there are individual differences and trial-level variability [57].

The simple detection task followed a within-subject design with sensory condition (visual, auditory, and multisensory) as the fixed factor. We extracted both descriptive statistics and inferential statistics in order to answer our three research questions. We used a GLMM as a more appropriate statistical model.

For question one, we used a GLMM with a gamma distribution with a log link function. Sensory condition was a fixed effect, and Participant was a random effect. For question two, a GLMM (gamma distribution with a log link function) was used to explore IIV across participants and across trials, modelling sensory condition as a fixed effect and Participant ID and Trial as random effects. Consistent with Barr et al. [56], random intercepts and slopes were included to account for baseline differences across participants and trials and to account for variability in the experimental effect across participants and trials, ensuring appropriate generalization and control of Type I errors. For question three, a GLMM (gamma distribution with a log link) explored differences in response times between the two auditory stimuli. The criterion for statistical significance was set at *p* ≤ .05 for all analyses reported in this study.

All valid RTs were included in the analysed data. A reaction time was considered valid when a response was made (i.e. a miss constitutes a trial when no response was made) and when the reaction time was between 0.15 and 1.0 s [58]. Reaction times that were faster than 0.15 s were considered anticipatory and faster than physiologically possible. RTs slower than 1.0 s were considered as lapses in attention. The number of misses as well as number trials with RTs outside of the valid range was quantified and compared across sensory conditions. Of the 6600 trials, 172 were excluded based on the above criteria (71 from the Auditory condition, 49 from the Audiovisual condition, and 52 from the Visual condition). Beyond trials excluded for being too quick or too slow, no other RTs were excluded as they were considered significant to our research question on intra-individual and interindividual variability.

To assess whether any reaction time facilitation exceeded predictions based on probability summation and thus whether there was evidence for behavioural integration prior to motor response initiation, we also computed the so-called Race Model inequality [58] for each participant. To do this, the cumulative distribution functions (CDFs) were calculated for each condition from each participant using bins of 10 ms width. We compared the CDF values from the multisensory condition (CDF_multisensory_) with modelled values based on the CDF from each of the unisensory conditions (i.e. race model prediction = [CDF_auditory_ + CDF_visual_- (CDF_auditory_*CDF_visual_)]). Any RT bin where the CDF values for the multisensory condition exceeded this model was taken as evidence for integration. Statistical testing at each bin was performed with a 1-sample *t*-test and a consecutive bin threshold of at least 5 bins (in order to account for multiple testing; see [29] for similar thresholding).

All analyses, with the exception of the race model inequality, were conducted in R [59]. Data preprocessing and manipulation were performed using the *tidyverse* [60]. GLMMs were fitted using *lme4* [61] *glmmTMB* [62], with post-hoc comparisons and estimated marginal means computed using *emmeans* [63]. Model diagnostics were conducted using the *performance* package. Data visualization was performed using *ggplot2* [64] and *sjPlot* [65]. Other packages used were *dplyr*, *broom*, *lmertest*, *tibble*, *tidyr*, *knitr*, *matrix*, *ggeffects*, and *afex* [66], [67], [68], [69], [70], [71], [72].

## 3 Results

### 3.1 Differences in central tendency of reaction times across sensory modalities

**Fig. 1:**
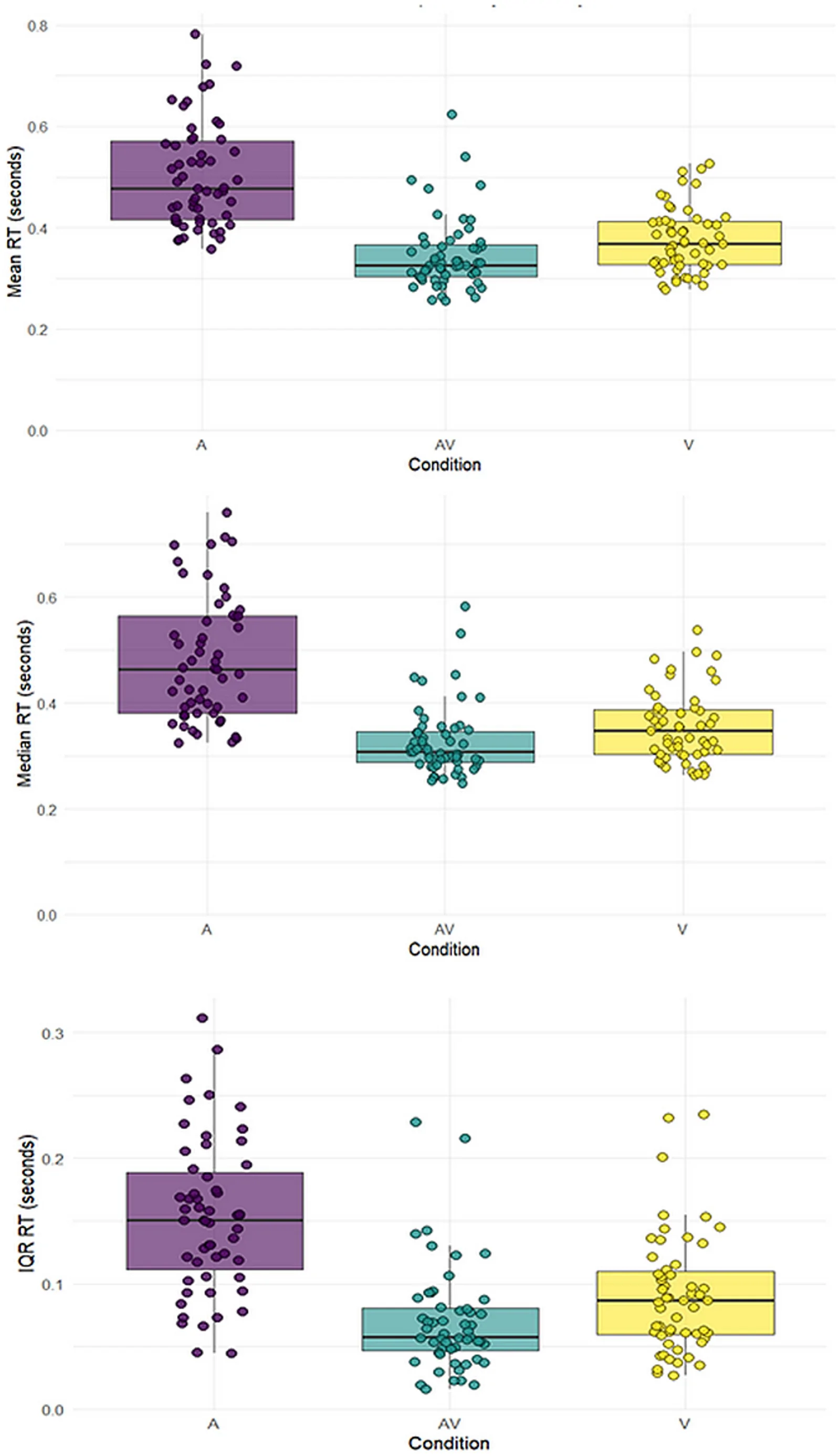
Aggregate measures of RTs. Boxplots and whisker plots display the mean, median, and interquartile range (IQR) of reaction times (top, middle, and lower panels, respectively) to auditory, audiovisual and visual stimuli (purple, green, and yellow, respectively). (For interpretation of the references to colour in this figure legend, the reader is referred to the web version of this article.)

We hypothesized that participants would demonstrate faster mean and median RTs as well as smaller IQRs to audiovisual multisensory stimuli than to either auditory or visual stimuli alone. Descriptive statistics (in seconds) indicated that RTs were slowest and most varied in the Auditory condition (mean = 0.503, median = 0.481, IQR = 0.153), were quicker and less varied in the Visual condition (mean = 0.375, median = 0.355, IQR = 0.091), and were the fastest and least varied in the Audiovisual condition (mean = 0.345, median = 0.327, IQR = 0.070). Boxplots and whisker plots show the distribution of the group-averaged mean, median and IQR of reaction times (Fig. 1A, B, and C, respectively).

Given the repeated measures and distribution of the RT data, a GLMM is a more appropriate statistical model. The GLMM (gamma distribution with a log link) was estimated using maximum likelihood and a Nelder-Mead optimizer to predict reaction times. 95% Confidence Intervals (CIs) and *p*-values were computed using a Wald t-distribution approximation. The model included Participant as a random effect and Sensory Modality as a fixed effect. Because the GLMM was fitted using a Gamma distribution with a log link, effects are first estimated on the log scale—ensuring positive fitted values and modelling multiplicative relationships with variance increasing with the mean—and are then back-transformed (exponentiated) to the original scale for interpretation.

The GLMM's total explanatory power was substantial (conditional R^2^ = 0.35), and the part attributable to the fixed effect alone was also substantial (marginal R^2^ = 0.28). On the log scale, the model's intercept, corresponding to when Sensory Condition is Auditory, was significantly shifted and negative (β_0_ = −0.71; 95% CI [−0.76, −0.65]). Within this model, the estimate for the Audiovisual condition was significantly shifted and negative (β = −0.37; 95% CI [−0.39, −0.36]; t_(6423)_ = −54.47; *p* < .001). Similarly, the estimate for the Visual condition was significantly shifted and negative (β = −0.29; 95% CI [−0.30, −0.27]; t_(6423)_ = −41.78; *p* < .001). For interpretability, marginal means and confidence intervals were then back-transformed from the log scale to the original RT scale. Back-transformed mean RTs were slowest for Auditory: 0.494 s (95% CI = [0.466, 0.523]), faster for Visual: 0.370 s (95% CI = [0.349, 0.393]), and fastest for Audiovisual: 0.339 s (95% CI = [0.320, 0.360]). Pairwise comparisons (Tukey-adjusted) indicated that all contrasts were significant: Auditory slower than Audiovisual: ratio = 1.454, SE = 0.01, z = 54.471, *p* < .001; Auditory slower than Visual: ratio = 1.333, SE = 0.009, z = 41.785, *p* < .001; Audiovisual faster than Visual: ratio = 0.917, SE = 0.006, z = −12.682, *p* < .001. Therefore, these results support our original hypothesis of RTs being fastest in the audiovisual (multisensory) condition. Model adequacy was evaluated using posterior predictive checks and residual diagnostics comparing the linear regression model to the GLMM (see Supplementary Fig. S2). These diagnostics supported the suitability of the gamma distribution.

### 3.2 Intra- and inter- individual Variability in Response Times Across Modalities

We next examined the extent to which there is significant intra- and/or inter- individual variability in RTs across trials on a simple detection task and the potential impact of the sensory modality of the stimulus. Our hypothesis was that IIV would also decrease under multisensory conditions. To this end, the data were fitted with a GLMM (gamma distribution with a log link) that was estimated using maximum likelihood and a Bound Optimization by Quadratic Approximation (BOBYQA) optimizer to predict reaction times. 95% Confidence Intervals (CIs) and *p*-values were computed using a Wald t-distribution approximation. The model included Sensory Condition as a fixed effect and both Participant and Trial (from 1st to 40th) as random effects. Random intercepts and slopes for participants and for trials were modelled as consistent with recommendations in Barr et al. [56]. This approach allowed simultaneous estimation of average Condition effects and individual differences in response patterns across trials.

This expanded model, like that above, exhibited a significant effect of Sensory Condition on RTs with audiovisual responses being fastest and auditory responses being slowest (see Table 1). Pairwise Tukey-adjusted comparisons confirmed significant differences: Auditory slower than Audiovisual (ratio = 1.457, SE = 0.031, z = 17.49, *p* < .001), Auditory slower than Visual (ratio = 1.336, SE = 0.034, z = 11.26, *p* < .001), and Audiovisual faster than Visual (ratio = 0.917, SE = 0.017, z = −4.59, *p* < .001).

**Table 1:** *Generalised mixed-effects model results for reaction times (*log *scale).*

Predictor / Modality	Β	SE	95% CI	z	P
Intercept (Auditory)	−0.708	0.053	[−0.813, −0.604]	−13.27	< 0.001
Audiovisual	−0.377	0.022	[−0.419, −0.334]	−17.49	< 0.001
Visual	−0.290	0.026	[−0.341, −0.240]	−11.26	< 0.001

**Fig. 2:**
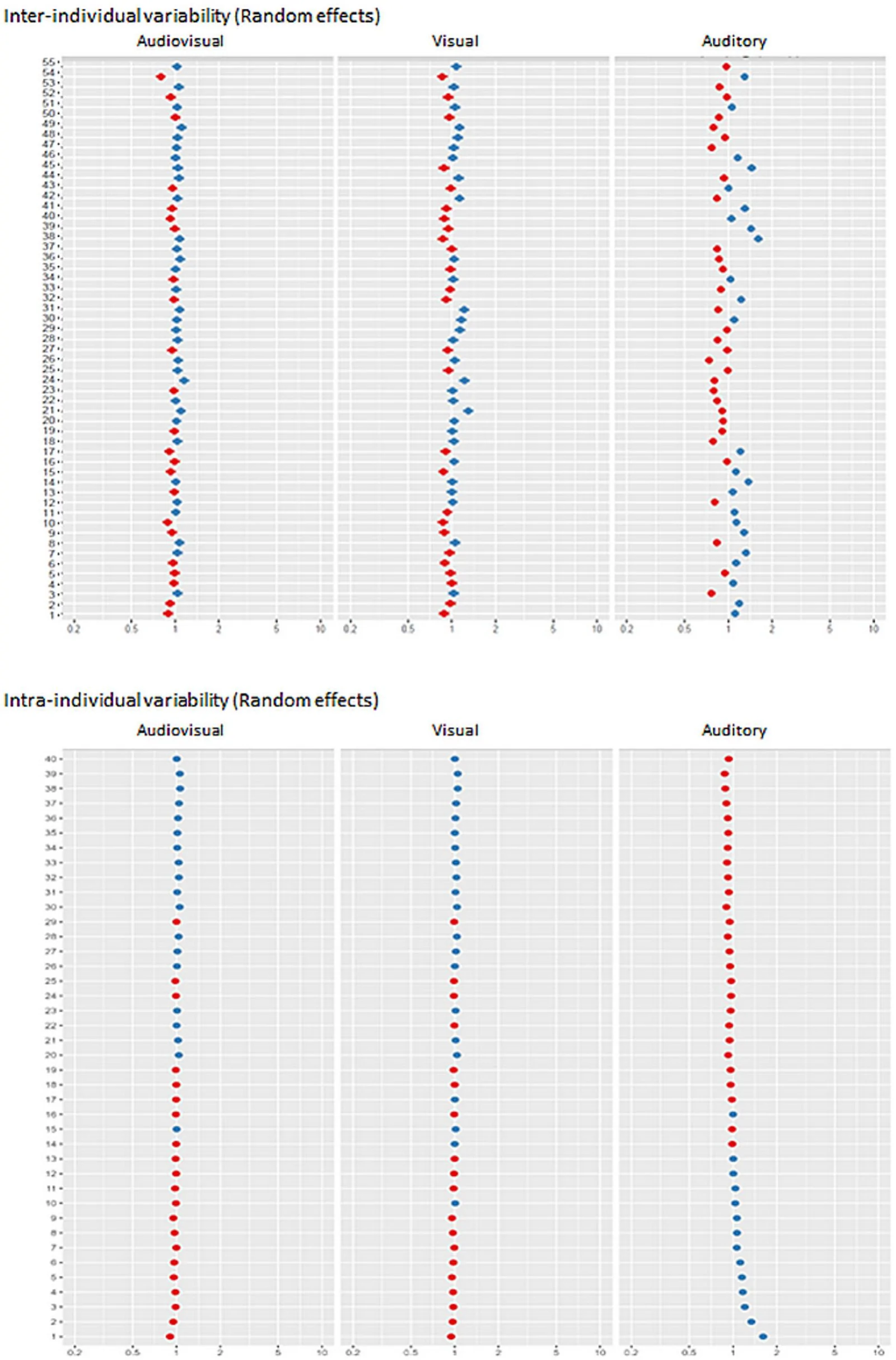
Behavioural variability. (top) Estimated random effects (participant-level deviations) from the GLMM. The numbers on the y-axis represent the participant numbers 1–55. Random effects close to 1 indicate values near the deviation from the fixed effect, while values above (blue) or below 1 (red) represent deviations from the overall mean response. (bottom) Estimated random effects (trial-level deviations) from the GLMM. The numbers on the y-axis represent trial numbers 1–40. Points represent the conditional modes (best linear unbiased predictions) of the random effects. Random effects close to 1 indicate values near the deviation from the fixed effect, while values above (blue) or below (red) 1 represent deviations from the overall mean response. (For interpretation of the references to colour in this figure legend, the reader is referred to the web version of this article.)

**Fig. 3:**
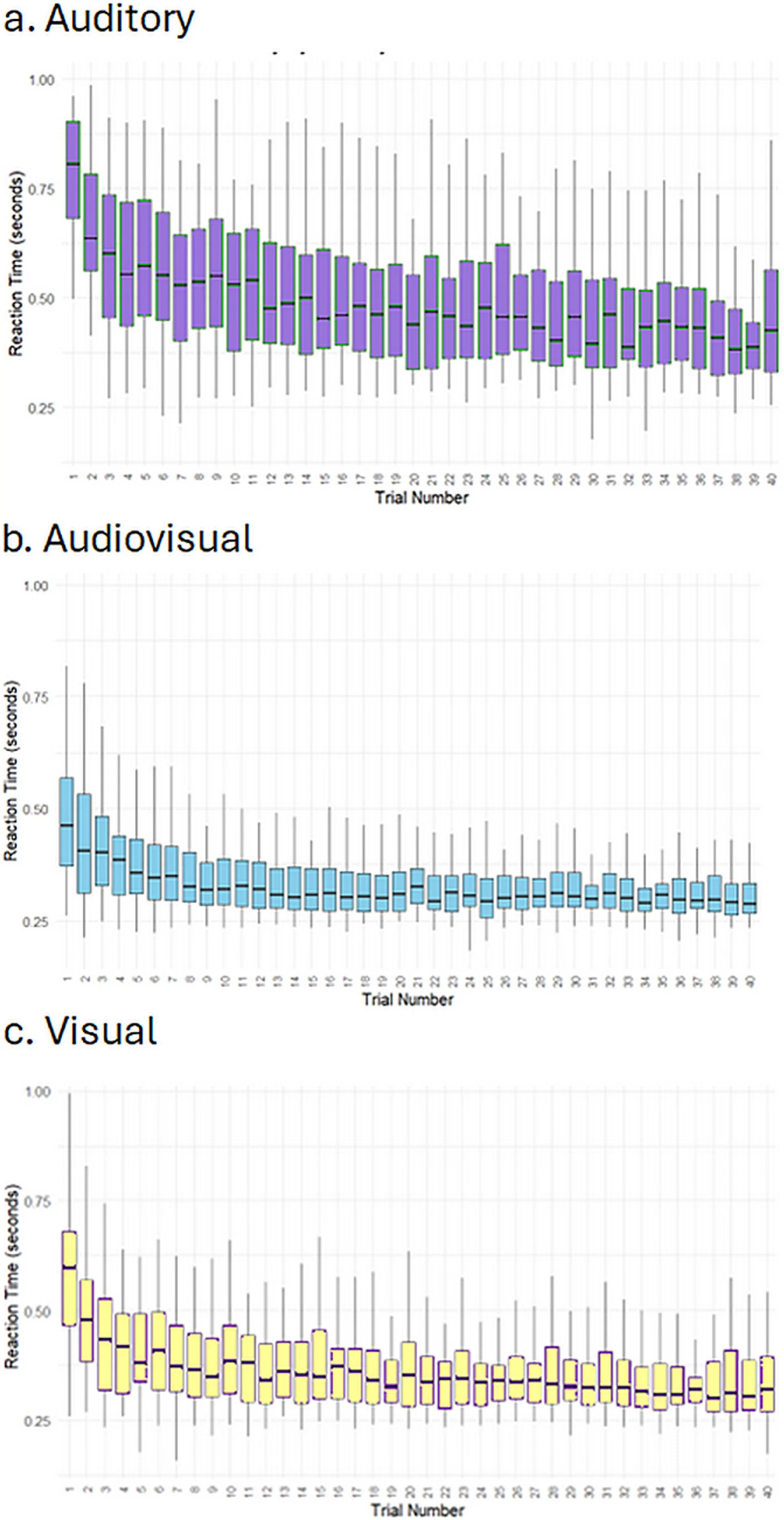
Inter-individual variability across trials and sensory conditions. Box and whisker plots of the interquartile range (IQR) across participants as a function of trial number per sensory condition. Trial-wise IQRs were largest for the auditory modality and smallest for the audiovisual condition. Across sensory modalities the IQR reached an asymptote within the first ∼10 trials.

In terms of inter-individual variability, there was moderate variability in the intercepts across participants (SD intercept = 0.091; SD slope AV = 0.065; SD slope V = 0.080). This variability provides an indicator of general RT differences across participants. Similarly, in terms of intra-individual variability there was also moderate variability in the intercepts across trials (SD intercept = 0.065; SD slope AV = 0.035; SD slope V = 0.032). This pattern suggests that both baseline reaction times and the effect of Sensory Condition on RTs varied across individuals as well as across trials. Audiovisual trials were the least variable both at the individual participant and at single-trial levels. Therefore, results support our hypothesis that there would be less variability in the audiovisual trials. Visualizations are presented on the normalised response scale and included both condition-level effects and random-effects displays to illustrate participant-level and trial-level variability (Fig. 2A and B, respectively). These visualizations suggest that participant-level variability is greater than variability at the trial-level across sensory conditions. Moreover, between visual, audiovisual, and auditory trials, the figures point to higher intra-individual and inter-individual variability in the auditory trials versus the other sensory conditions. We also explored the group-level IQR across the 1st to 40th trial for each sensory condition (Fig. 3). These visualizations also suggest higher IIV in the auditory and visual conditions than in the audiovisual condition. What is more, the IQR for each modality appears to asymptote within the first ∼10 trials and to remain relatively stable over the duration of the experiment, providing no indication of major learning-related or fatigue-related performance changes.

### 3.3 Race model

**Fig. 4:**
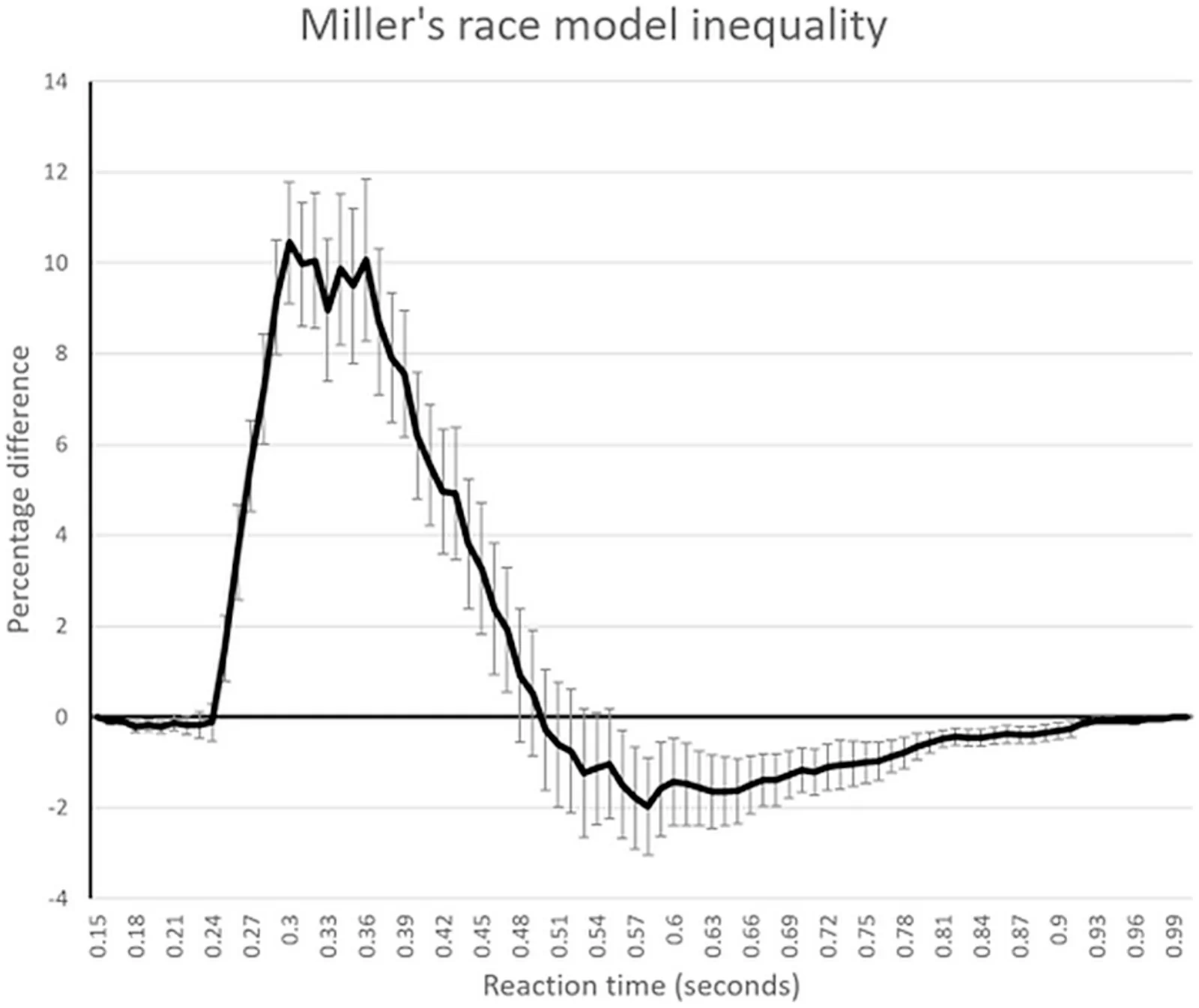
Multisensory facilitation of RTs beyond race model predictions. The difference between the observed and modelled CDFs are displayed as a function of reaction time. The percentage difference is displayed on the y-axis, and positive values indicate facilitation. Statistical tests (1-sample *t*-tests) were performed across RT bins (0.01 s width) with the criterion of at least 5 contiguous bins exhibiting a significant difference. Significant effects were observed over the 0.25–0.45 s RT range.

The RT data from each participant and stimulus condition were transformed into CDFs that were compared using Miller's race model inequality. Over the 250-450 ms RT range, there were significant violations of the race model inequality, indicating that the facilitation observed in the audiovisual condition exceeded predictions based on probability summation alone and suggestive of integrative processes prior to motor response initiation (Fig. 4). The magnitude of this multisensory gain reached ∼10%.

### 3.4 Pitch-related differences in reaction times

Our hypothesis was that RTs to higher-pitched sounds would be significantly faster than those to lower-pitched sounds. To assess whether response times differed as a function of the auditory pitch of the stimulus, analyses were restricted to the auditory trials. As a reminder, two sounds were used in the simple detection task; the “yawn” stimulus had peak energy in lower frequencies (i.e. <500 Hz), whereas the “whistle” had peak energy in higher frequences (i.e. ∼2000 Hz) (see Supplementary Fig. S1).

**Fig. 5:**
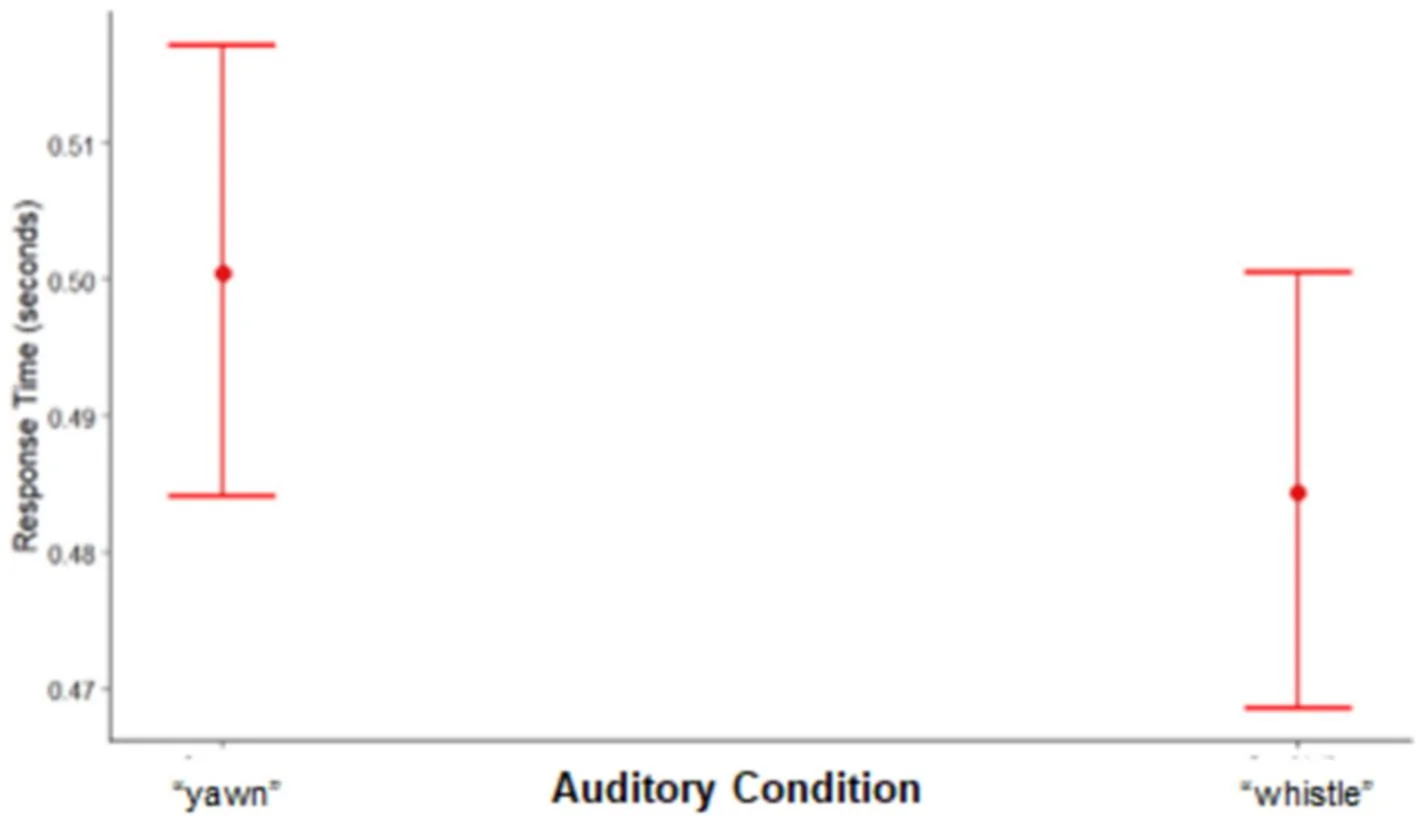
Back-transformed RTs for the two auditory stimuli. Points represent the estimated mean response time for the two auditory conditions. Error bars indicate the 95% confidence interval for the predicted values.

The data were fit with a GLMM (gamma distribution with a log link) that was estimated using maximum likelihood and a Nelder-Mead optimizer to predict reaction times. Predictive checks supported the model fit of the GLMM. 95% Confidence Intervals (CIs) and *p*-values were computed using a Wald t-distribution approximation. The model included Participant and Trial as random effects and Pitch as a fixed effect. The model's total explanatory power was moderate (conditional R^2^ = 0.15), and the part related to the fixed effect alone (marginal R^2^) was 0.003. On the log scale, the model's intercept, corresponding to when Pitch was the “yawn”, was at −0.69 (95% CI [−0.76, −0.62]). The estimate for when Pitch was the “whistle” was significantly shifted and negative (β = −0.03; 95% CI [−0.05, −0.01]; t_(2125)_ = −3.08; *p* = .002). Marginal means and confidence intervals were back-transformed from the log scale to the original response scale. Back-transformed mean response times were slower for “yawn” 0.500 s, 95% CI [0.467, 0.536] and faster for “whistle” 0.484 s, 95% CI [0.452, 0.518] (Fig. 5). Pairwise comparisons (Tukey-adjusted) indicated that the “yawn” and “whistle” sounds differed significantly (ratio = 1.033, SE = 0.011, z = 3.085, *p* = .002). Therefore, our results support our hypothesis of a significant difference between the RTs to the two auditory stimuli. We also examined whether this difference persisted on multisensory trials, regardless of the specific visual stimulus pairing. We used a GLMM as described above. Back-transformed mean response times were slower for multisensory conditions that included the “yawn” sound 0.342 s, 95% CI [0.320, 0.365] and faster for multisensory conditions that included the “whistle” sound 0.336 s, 95% CI [0.315, 0.358]. Pairwise comparisons (Tukey-adjusted) indicated these differed significantly (ratio = 1.02, SE = 0.008, z = 2.246, *p* < .025). When applying a similar analysis to the visual conditions, no reliable difference was observed (*p* > .90). Finally, we calculated the percent multisensory benefit on median reaction times separately for those conditions involving the “yawn” and “whistle” auditory stimuli. Using a 2-tailed paired *t*-test, there was a non-significant trend for larger benefits for conditions involving the “yawn” than “whistle” (7.2% vs. 5.3%; t_(54)_ = 1.75; *p* = .085), which is consistent with expectations based on the principle of inverse effectiveness (e.g., [73]).

### 3.5 Exploring links between variability and multisensory benefits

We explored whether intra-individual variability in reaction times, as measured by IQR, was associated with the magnitude of multisensory benefits. To do this, we calculated the Pearson correlation between the IQR from each participant and their percent multisensory benefit, which was calculated as the percent difference in median reaction times between the faster unisensory and multisensory conditions. There was a significant positive correlation between the IQR on the auditory trials and percent multisensory benefit (r_(53)_ = 0.348; *p* < .01). This was also observed for the IQR on the visual trials (r_(53)_ = 0.467; *p* < .0005). No significant correlation was observed using IQR on the multisensory trials (r_(53)_ = −0.145; *p* > .29).

## 4 Discussion

We replicated and extended the findings of multisensory gains on reaction times by healthy older participants. To this end, 55 cognitively healthy older participants completed a simple detection task with auditory, visual, and audiovisual stimuli. Consistent with prior work [8], [29], we found that response times to multisensory stimuli were significantly faster than those to either the visual or auditory stimuli, with the auditory condition being significantly slower than each of the two other conditions. We also extended the findings of Murray et al. [8] by exploring the question of variability at both inter-individual and intra-individual levels. We observed significant inter-individual and intra-individual variability that was itself modified by stimulus condition. Specifically, and consistent with our hypothesis, the variability of response times was significantly smaller with multisensory stimuli compared to either auditory or visual stimuli, and the variability of response times to auditory stimuli was the largest. Lastly, we explored if there were RT differences between the two pitches used in our paradigm. Our results support our hypothesis, pointing to a significant difference in RTs between the two pitches. In what follows, we first discuss multisensory processing, then variability, and finally within-modality processing. We then consider some limitations and discuss the outlook of this work.

Our results align with a substantial body of work demonstrating multisensory gains using simple reaction time paradigms [8], [11], [12], [26]. Such effects have been observed across the lifespan, including in schoolchildren, young adults, as well as the elderly [8], [27], [48]. Multisensory gains are thought to reflect enhanced efficiency of perceptual processing that allows for faster accumulation of sensory evidence and more rapid motor responses (e.g. [26], [74]). In our results, RTs to multisensory stimuli were ∼ 8–10% faster than those to visual stimuli. This percentage gain, which was also observable in the race model analysis (Fig. 3) is highly comparable to values reported in Murray et al. [8] for both healthy older and younger participants, as well to values reported in 9-year-old children [48].

Our observation of slower RTs to auditory compared to visual stimuli is also consistent with several previous reports in older adults, potentially reflecting age-related changes in auditory processing speed, sensory reliability, or attentional allocation [8], [11], [28], [75], [76]. These perceptual changes have been shown to affect cognitive function [77], and some research has suggested that auditory changes can be considered as precursors to cognitive decline [78], while other research has argued that there could be a common cause between auditory changes and cognitive decline [10], [16], [79]. While no explicit effort was undertaken here to equate the efficacy and saliency of the auditory and visual stimuli, both were clearly supra-threshold in terms of processing sensitivity. That is, the auditory stimuli were comfortably loud and presented against a silent background, and the visual stimuli were 100% contrast presented against a black background. Nonetheless, it must be acknowledged that visual stimuli had an abrupt onset, whereas the auditory stimuli unfurled in time. Such notwithstanding, we would note that detection accuracy was near ceiling, with only 2.6% of trials excluded from analyses, including for reasons of anticipation and unusually slow reaction times that may be due to momentary lapses of attention. What is more, the significant reaction time difference between multisensory trials containing different sounds cannot be explained by such saliency or onset characteristics. Overall, our results support previous findings that auditory perception may be weaker than visual perception in healthy older adults without cognitive decline [8], though this will require more systematic and parametric investigation in future research.

Furthermore, we were also able to extend the literature on multisensory processing in ageing by looking at intra-individual variability across trials and also across participants. Our results revealed significant variability both between individuals (inter-individual variability) and within individuals across trials in the auditory condition, whereas in the multisensory condition variability was significantly lower. Such variability was evident at both the participant-level and the trial-level, highlighting potential attentional and sensory processing mechanisms in cognitively healthy ageing when confronted with auditory stimuli alone or along with visual stimuli. Results suggest that the auditory condition not only elicits the slowest response times but also shows the highest variability at an inter-individual level and at an intra-individual level. These results fill a gap in the literature, which has studied RTs at an aggregate level or compared one population with another without considering trial variability. The fact that auditory RTs are the slowest as well as show the highest variability across trials suggests that auditory processing may be particularly sensitive to underlying cognitive processes and early declines in neural efficiency, potentially making it a valuable behavioural marker for detecting subtle cognitive changes [80]. However, the lower and less variable RTs on multisensory conditions suggests that multisensory integration may not only enhance processing speed but also make attention more stable [34], [41]. Some research using EEG in young adults has suggested that this may reflect more robust neural processing or more reliable attentional engagement than when auditory and visual modalities are presented alone [49]. Variability within individuals was associated with changes in coordinated activity spanning an occipital-to-frontal network [49]. In young adults, differences in RT variability between individuals were related to varying levels of engagement in the medial frontal cortex [49]. Although we are unable to make any physiological claims from our results, our results could be seen to complement a growing body of work pointing to the measure of IIV as being important in contributing our understanding of cognitive performance, attention, and impulsivity especially as it relates to ageing [33]. In this vein, exploratory analyses further indicated that within-subject variability, as measured via IQR, was positively correlated with the percentage of multisensory benefits on median RT. This was the case both for IQR from the unisensory auditory and unisensory visual conditions but was not observed when testing IQR from the multisensory condition. This pattern is consistent with models of multisensory processing that emphasize stimulus reliability; sometimes framed as the principle of inverse effectiveness (e.g. [73]). It is perhaps important to emphasize that such models refer to percentage rather than absolute gains, wherein situations of reduced reliability/effectively (by extension increased variability as measured by IQR) result in the largest percentage gains, even if not the absolutely fastest or most intense response. The present results thus suggest that such models can be assayed using reaction time data from individual participants.

Given that increased IIV has been linked to attentional lapses, reduced cognitive control, and early cognitive decline, the present findings suggest that using a simple detection tasks could provide a framework for exploring subtle cognitive changes in ageing and in sensory processing [32], [35], [36], [49], [57]. In a review of 22 studies, higher than baseline IIV was seen as a precursor to dementia and cognitive decline and falls [34], [80]. Lastly, research has shown that measuring IIV in cognitive performance can be more informative than using standard cognitive performance measures, such as the MoCA, that cannot assess IIV. Instead, IIV may provide better information regarding an individual's cognitive health and potentially provide early signs of cognitive decline [36], [80], [81], [82]. Finally, our results are important as research has shown that variability can be improved with treatment. For example, research has shown that cognitive training and physical exercise can improve IIV [83]. Therefore, detecting high levels of IIV and implementing a treatment plan is important in order to help reduce the effects of increased IIV [84]. Using simple detection tasks, as part of the traditional cognitive tests could provide important information to clinicians in order to detect subtle cognitive changes that are undetectable via traditional neuropsychological tests such as high levels of IIV [80]. This could allow for preventative treatment plans to be implemented at the early stages of cognitive decline [33]. Our results in terms of IIV can therefore be seen as filling a potentially important gap in the literature.

Beyond IIV, we were also able to examine differences in stimulus processing within a sensory modality. Participants demonstrated systematically different RTs as a function of stimulus pitch, indicating that IIV in the auditory condition cannot be attributed solely to fluctuations in attention. Rather, these findings point toward pitch-dependent perceptual and neurocognitive mechanisms that modulate information processing speed [85]. In a study using the same paradigm and sounds in schoolchildren, no significant difference was found between the two sounds [48]. Instead, our results suggest subtle changes may be occurring with age. Research has shown that even in cases where there is no detectable presbycusis, both high frequency tones and low frequency tones can be difficult to detect due to age-related neural degeneration both at the level of the cochlea and central auditory system [86][87]. Other research has shown that ageing appears to generally increase neural responsiveness, versus responses in young adults, to a similar degree in individuals with and without presbycusis, which may reflect neurophysiological compensation [88]. While these changes may account for general age-related slowing, they do not readily account for the differences we observed between stimuli. One possibility is that ageing is functionally affecting some pitch representations more than others. There is generally an over-representation of neural populations sensitive to pitch ranges of human speech between ∼20 Hz and 2 kHz [89], [90]. With ageing, there is evidence that these neural populations become more broadly tuned [91] albeit similarly organized in a tonotopic manner [92], which could translate in slower and more variable responsiveness and RTs, particularly for stimuli with pitches covering this range. An alternative possibility is that the “whistle” is simply more startling than the “yawn” given its higher peak frequency (see also [93] for evidence of preserved startle responses to sounds in the elderly). However, other research suggests that the elderly perceive lower frequencies with more urgency [94]. As the present study was not specifically designed to identify stimulus-related factors affecting RTs, we cannot provide an unequivocal interpretation of the difference we observed. However, the present findings underscore the necessity of explicitly accounting for low-level stimulus characteristics when interpreting response time variability, particularly in ageing research. Lastly, these results warrant further investigation in how simple detection tasks can facilitate the characterisation of pitch detection differences, particularly in ageing [95].

Collectively, the present findings contribute to mounting evidence suggesting that simple detection tasks could potentially offer a promising avenue for understanding attentional factors, sensory integration, and cognitive health in older adulthood as well as potentially detect and monitor subtle changes in order to offer better preventative treatments or complement diagnostics [8]. Our results are consistent with theoretical accounts positing that degraded sensory input, particularly in the auditory system, places strain on higher-order cognitive systems [10], [13]. Furthermore, the fact that multisensory stimuli elicit the fastest and least variable response times highlights the need to take this into consideration whether when designing systems to notify pedestrians when it is safe to cross streets or for use in other contexts that require sustained attention in busy environments. Lastly, given that high IIV is considered a risk for falls and could be a precursor to cognitive decline, it would be important to explore ways to decrease IIV and increase sustained attention preventatively or before cognitive decline is apparent.

### 4.1 Limitations and outlook

This study has several limitations. There is no comparison group, nor have we considered age as a covariate in our analyses. However, there was little variability in the age range of the participants in this study. The difference in reaction times to the two different pitches would need to be explored further to see if these differences would be seen with other pitch sounds. Furthermore, as the study depended on self-reported confirmation of no hearing loss, it would be important to confirm this through audiological tests. Lastly, this study only used a small portion of the data collected for this larger project for which data collection remains ongoing. It will be important to compare the results from the simple detection task with the data collected through MRI/fMRI, the larger battery of neuropsychological tests, as well as other psychophysics tests that were collected. Future research could also explore whether reduced variability in multisensory contexts generalizes to more complex cognitive tasks and whether such effects are maintained in populations at risk for cognitive decline. Furthermore, longitudinal studies may also help determine whether auditory response times and variability measures could serve as early indicators of cognitive decline.

## 5 Conclusion

Overall, our findings support and extend the literature on multisensory gains and auditory processing in response time paradigms. Importantly, by incorporating analyses of intra-individual variability, the present study advances understanding of how different sensory modalities are affected by IIV, in particular auditory and multisensory conditions. Although further studies are needed to explore how auditory pitch affects response times, the observed pitch-related differences highlight the potential value of fine-grained auditory manipulations in probing sensory–cognitive interactions in ageing and suggest that auditory processing changes, and not simply attentional changes, affect response times. By characterising intra-individual variability and (multi)sensory processing during healthy ageing, this study improves the understanding of sensory processing across the lifespan, extending beyond prior works in young adults and children and moving toward statistically more robust analyses. In so doing, this work also highlights the utility of a simple detection task in functional assessments.

## Declaration of generative AI use

The authors declare that generative AI was not used in the preparation of this manuscript or in data processing/analyses.

## Studies in human

This study was performed in compliance with relevant laws, regulatory frameworks and guidelines where the research took place.

This study was approved by the Cantonal Ethics Committee of the Canton of Vaud.

(Approval No. 2024–00584)

## CRediT authorship contribution statement

**Naomi K. Middelmann:** Writing – review & editing, Writing – original draft, Visualization, Supervision, Project administration, Methodology, Investigation, Funding acquisition, Formal analysis, Data curation, Conceptualization. **Lauriane Ecabert:** Writing – review & editing, Project administration, Investigation, Data curation. **Eduardo Villar Ortega:** Writing – review & editing, Software. **Alessandra Griffa:** Writing – review & editing. **Andrea Brioschi-Guevara:** Writing – review & editing, Investigation. **Gilles Allali:** Writing – review & editing, Investigation, Conceptualization. **James E. Bartlett:** Writing – review & editing, Visualization, Supervision, Formal analysis. **Micah M. Murray:** Writing – review & editing, Writing – original draft, Supervision, Project administration, Methodology, Investigation, Funding acquisition, Formal analysis, Data curation, Conceptualization.

## Informed consent and patient details

Written informed consent to take part in the study and to publish the article has been obtained from all participants or their legal representatives. The privacy rights of participants have been observed.

## Declaration of competing interest

The authors declare that they have no known competing financial interests or personal relationships that could have appeared to influence the work reported in this paper.

## Data Availability

The data used in this work are available from the corresponding authors upon reasonable request. The analysis code is available at: https://github.com/naomimiddelmann/Multisensory-Variability-SRT
