# Supplementary material for: Inter- and intra- individual variability in audiovisual multisensory processes in healthy ageing
Source: Aging Brain. 2026 Sep 14;10:100168. doi: 10.1016/j.nbas.2026.100168 (PMC13594627; doi:10.1016/j.nbas.2026.100168)
Supplement: Supplementary file 1 [file mmc1.pdf]

Supplementary Material

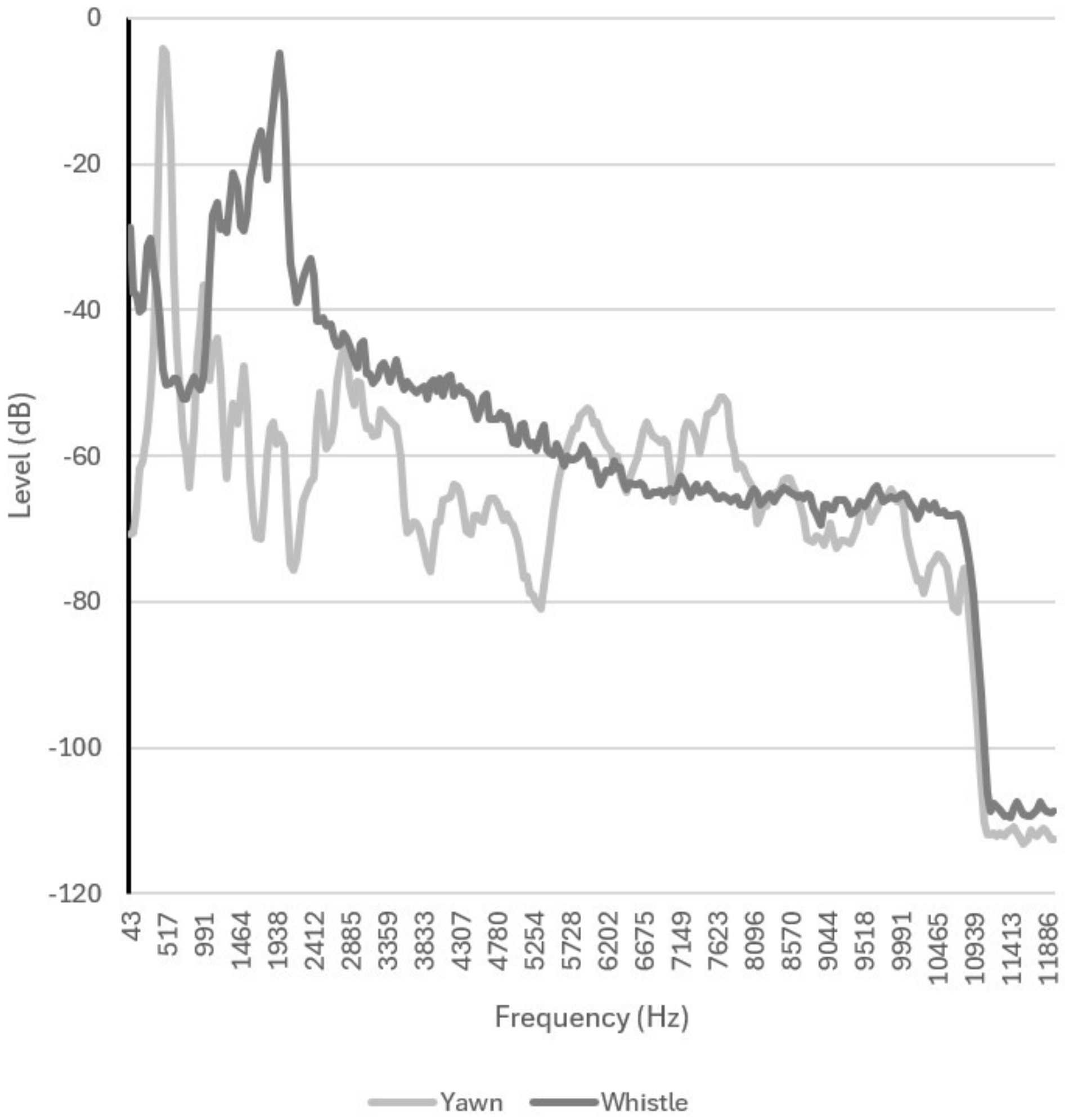

Supplementary Figure S1. Spectra of the two auditory stimuli indicates a larger intensity of lower frequencies for the "yawn" and larger intensity of higher frequencies for the "whistle".

Supplementary Material

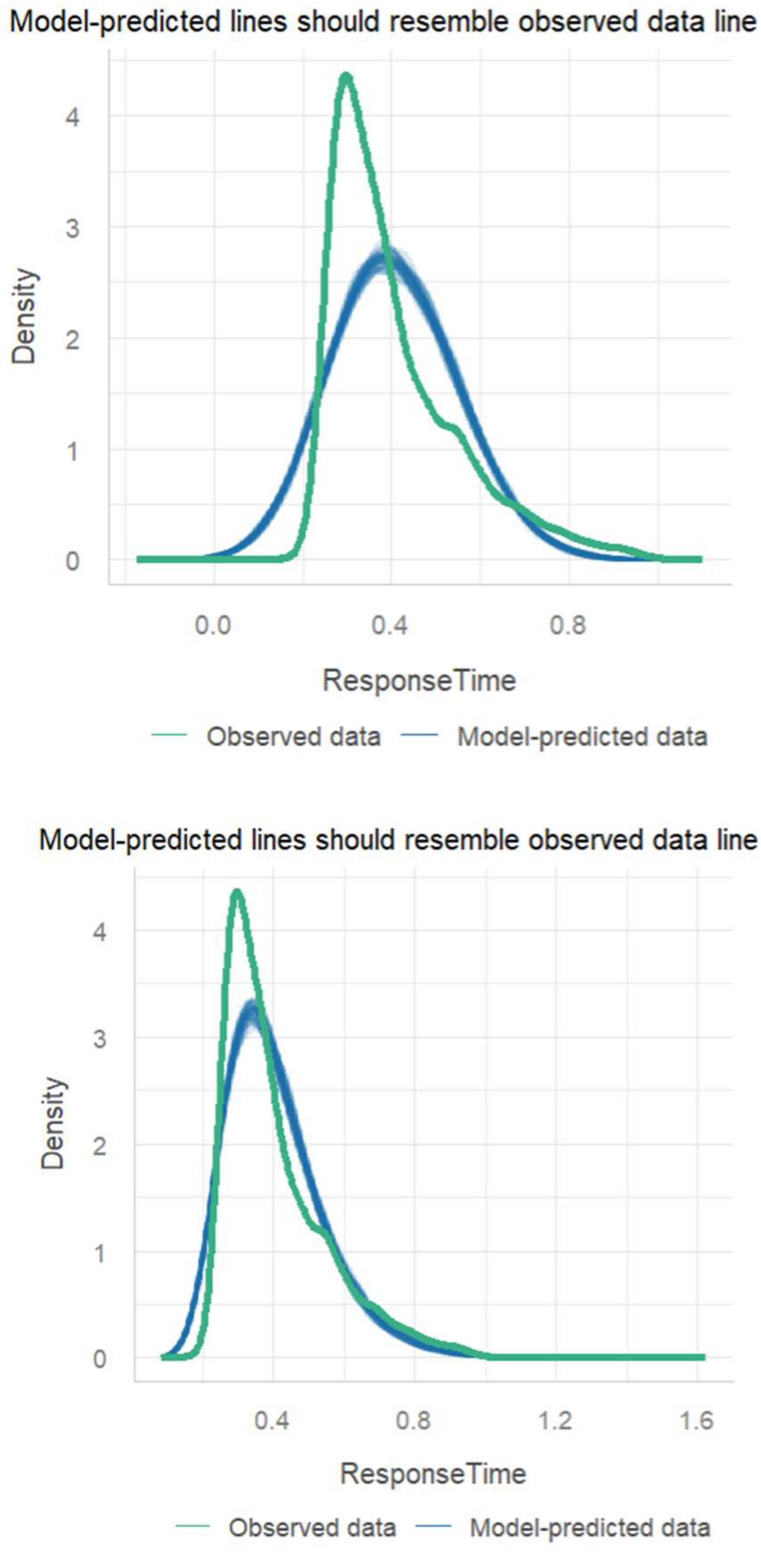

Supplementary Figure S2. Poster predictive check of the model fit of the simple linear regression (upper panel) and the GLMM using a gamma distribution with a log link (lower panel). In the case of the GLMM there is a closer alignment between the observed and modelled values.
